# Trends and predictors of changes in modern contraceptive use among women aged 15–49 years in Tanzania from 2004–2016: Evidence from Tanzania Demographic and Health Surveys

**DOI:** 10.1371/journal.pone.0234980

**Published:** 2020-06-29

**Authors:** Mashavu H. Yussuf, Bilikisu R. Elewonibi, Martin M. Rwabilimbo, Innocent B. Mboya, Michael J. Mahande

**Affiliations:** 1 Department of Epidemiology and Biostatistics, Institute of Public Health, Kilimanjaro Christian Medical University College, Moshi, Tanzania; 2 Harvard T.H. Chan School of Public Health, Boston, Massachusetts, United States of America; 3 School of Mathematics, Statistics & Computer Science, University of KwaZulu-Natal, Pietermaritzburg, Scottsville, South Africa; University of Botswana, BOTSWANA

## Abstract

**Introduction:**

Modern contraceptive use provides opportunities for women and couples to achieve optimal child spacing, achieve desired family size and reduce unsafe abortions. Despite these facts, modern contraceptive prevalence rate (mCPR) in Tanzania remains as low as 32%. This study aimed to determine trends and factors associated with changes in modern contraceptive use among women of reproductive age in Tanzania from 2004–2016.

**Methodology:**

This was a cross-sectional study utilizing data from Tanzania Demographic and Health Surveys of 2004–2005, 2010 and 2015–2016. Data analysis was performed using Stata version 14. Analysis considered the complex survey design through application of weights, clustering and strata. Multivariable Poisson decomposition analysis was used to assess factors associated with changes in modern contraceptive use. Results were presented in the form of decomposition coefficients and percentages.

**Results:**

Modern contraceptive use increased from 23.0% in 2004 to 34.3% in 2016. Differences in women’s characteristics contributed 12.5% of the increase in mCPR. These characteristics include partner’s education levels, recent sexual activity and being visited by a family planning worker. The difference in coefficients contributed 87.5% increase in mCPR. The most increase in modern contraceptive use was attributed to rural population (44.1%) and women who experienced a termination of pregnancy (7.1%).

**Conclusion:**

Modern contraceptive use has steadily increased in Tanzania. Health policies and interventions need to target sexually active women, rural residents as well as less educated women and men to maintain and further accelerate the trends in mCPR. Interventions focusing on women who experienced a termination of pregnancy may also serve as an entry point to promote use of modern contraceptive methods.

## Introduction

Modern contraceptive use helps women and couples to take charge of their fertility, achieve their desired family size and optimum child spacing [1–4]. Modern contraceptive use also helps to improve child survival by lengthening birth intervals thereby reducing sibling competition for scarce family resources [5]. Modern contraceptives prevent unwanted pregnancy, they also reduce a need for abortions, particularly unsafe abortions [4,6]. Furthermore, barrier methods such as condoms reduce the rate of HIV and sexually transmitted infections [4,7]. Modern contraceptives create opportunities for women to make informed choices about their reproductive and sexual health thereby enabling them to pursue educational advances and careers [2,4]. In Tanzania, it was estimated that the current modern contraceptive prevalence rate (mCPR) of 32% has averted 1,054,000 unintended pregnancies, 313,000 unsafe abortions and 3,000 maternal deaths in 2017 [8]. In Tanzania, modern contraceptive use has been increasing steadily from 20.0% in 2004/2005 to 27.4% in 2010 and further to 32% in 2015/2016 at an annual increase of 1.2% [9]. Despite the effort to achieve the national mCPR target of 45% by 2020, there are vast zonal variations in mCPR across the country with the highest mCPR in Southern Zone (50.5%) to lowest mCPR in Zanzibar (14.0%) [9].

The observed increase in mCPR could be due to changes in the population structure such as increase in the number of educated women and urbanization or due to changes in effects of certain exposure variables that are known to influence modern contraceptive use [10,11]. A previous study in Ethiopia reported that, changes in the population structure contributed to 34% of the overall change in contraceptive use from 2000 to 2011, particularly increase women’s educational attainments and family size concordance between couples [9]. Authors noted that after controlling for changes in the population structure, most (66%) of the changes in contraceptive use was due to the effects of some characteristics such as changing of contraceptive behaviour among rural population, Orthodox Christians and Protestants [10]. A study in Rwanda also reported an increase of mCPR from 17% to 52% between 2005 and 2010, where 77% increase in mCPR was due to changes in effects while the changes in population structure accounted only 17% of the changes in mCPR [10]. The variables which were significant to changes in effects included women’s education, experience of child mortality and place of residence. Significant changes in the population structure were seen in women’s education, exposure to FP messages in the media and family size concordance between couples [11].

Among the major social and economic challenges facing Tanzania at the moment is the high rates of population growth [12]. The National Bureau of statistics has projected the population of Tanzania will be 65 million by 2025, at the current annual growth rate of 2.9% [12]. These projections are attributed to high fertility rates of 5.2 births per woman and early initiation of child bearing [12,13]. If this will be left unchecked, it will threaten to further strain and overload the fragile health and educational services, infrastructure, food supply and the environment [12]. The implementation of family planning is among the top health priorities in Tanzania [12,14–16]. National efforts concerning family planning include the launching of the National Family Planning Costed Implementation Program, 2010–2015 which targeted a national CPR of 60% by 2015 [12]. The Ministry of Health and Social Welfare has also updated the National Family Planning Research Agenda (NFPRA) as an attempt aimed at identifying current gaps in family planning through evidence-based knowledge [14]. Further commitments are included in One Plan II which targets to achieve a national mCPR of 45% by 2020 and reduce the unmet need of family planning to 10% by 2020 [15]. The Tanzanian government has also targeted to double the number of FP users to 4.3 million by 2020 as part of FP2020 initiatives [16]. Despite these interventions, the country is far from achieving the national target of 45% mCPR by 2020. At the current 1.2% annual increase in mCPR, it will take 11 years for Tanzania to reach the 45% mCPR target.

It remains unclear what influences the changes in contraceptive dynamics in Tanzania. This study aimed to determine the trends and factors associated with changes in modern contraceptive use among women aged 15–49 years in Tanzania from 2004 to 2016. By disentangling the percentage contribution of each factor, areas in need of more focus may be clarified, thereby allowing for targeted evidence-based interventions to be made in order to maintain the increasing trends in modern contraceptive use in Tanzania.

## Methodology

### Study design and study area

This was an analytical cross-sectional study conducted using nationally representative secondary data from the Tanzania demographic and health surveys (TDHS) of 2004/2005, 2010 and 2015/2016. The study included data from all regions of the United Republic of Tanzania. The Tanzania DHS employs a multi-stage sampling procedure. The first stage involves a selection of a stratified sample from a list of enumeration areas (EAs) that have been obtained from the recent census conducted in Tanzania. These EAs are the clusters. This sample of EAs is selected with considerations to probability proportional to size (PPS) that takes into account the size of the enumeration area. At the second stage, after a complete list of households is available in each of the selected EAs, a fixed/variable number of households is selected by equal probability systematic sampling technique.

### Study population and sample size

**Fig 1:**
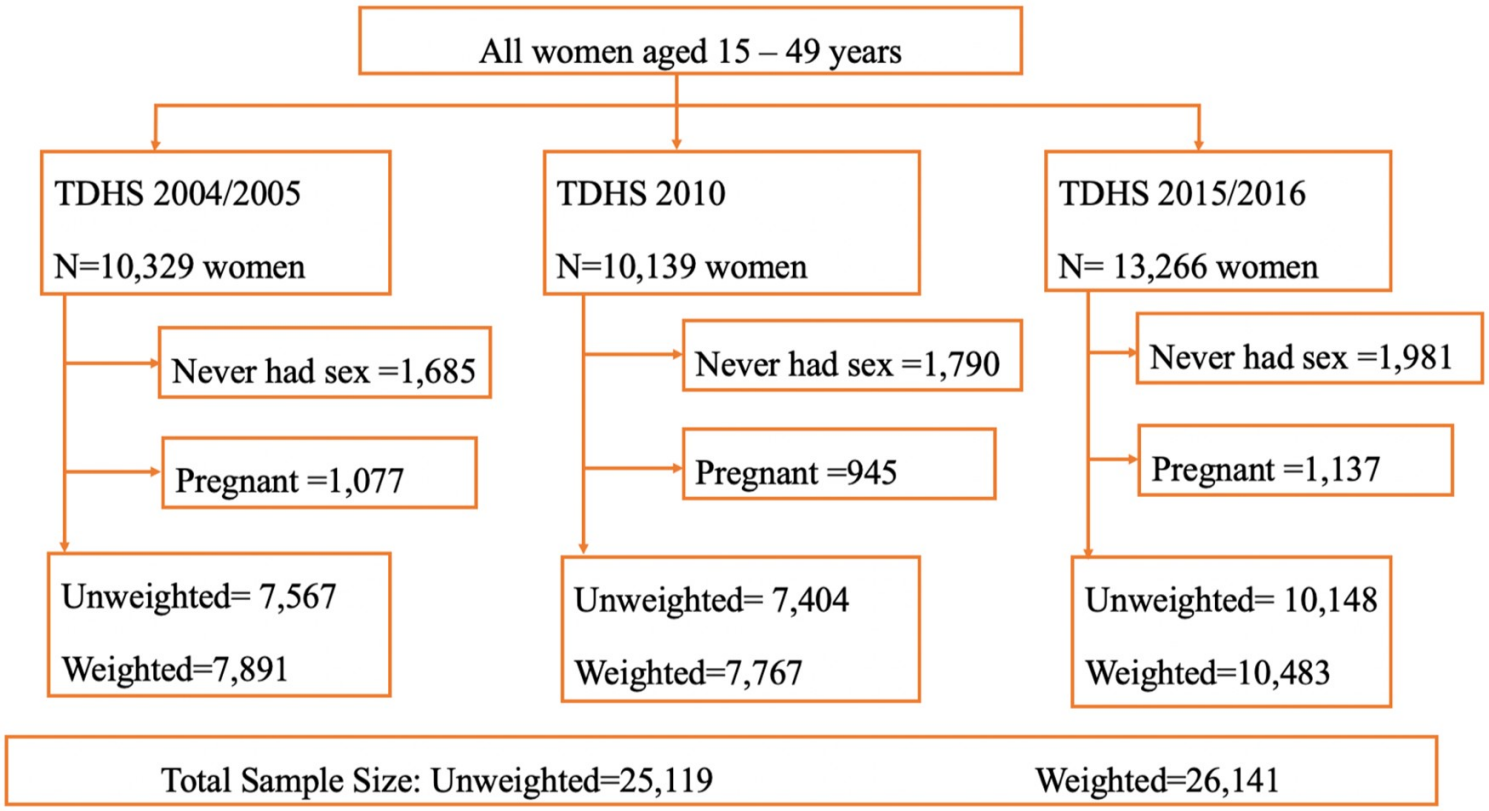
Flow chart for selection of study participants.

The study participants were restricted to non-pregnant women who have had sexual intercourse at the time of survey since they were at risk for an unintended pregnancy. Women who were pregnant at the time of survey as well as those who reported to never being sexually active were excluded since they were not at risk for pregnancy. This resulted in a total weighted sample of 26,141 from the three TDHS; 7567 women in 2004/2005 (7,891 weighted cases), 7,404 women in 2010 (7,767 weighted cases) and 10,148 women in 2015/2016 (10,483 weighted cases) (Fig 1).

### Measures

The outcome of interest in this study was modern contraceptive use, a binary variable being Yes if a woman was currently using a modern contraceptive method and No if a woman was not currently using a modern contraceptive method. Modern contraceptive methods for this study include male and female sterilization, injectables, intrauterine contraceptive devices (IUCDs), oral contraceptive pills, implants, male and female condoms, diaphragm, emergency contraception and lactational amenorrhea method (LAM).

Predictor variables in this study include both socio-demographic as well as reproductive health characteristics. Socio-demographic characteristics include: age of the woman in years [15–24, 25–34, 35–49], male-female age difference [woman is older, same age, man older by 9 years/less, man older by more than 9 years], geographical zones [Western, Northern, Central, Southern Highlands, Southern, South West Highlands, Lake, Eastern, Zanzibar], residence [Urban-rural], woman’s education level [No education, Primary, Secondary and above], partner’s education level [No education, Primary, Secondary and above], number of living children [None, 1–2, 3–4, 5 or more], wealth categories [Poor, middle, rich] and FP media exposure [No, yes]. Reproductive health characteristics include: Last pregnancy history [Wanted, mistimed, unwanted], household visit from FP field worker [No, yes], family size concordance [Both want same, husband wants more, husband wants fewer, don’t know], sexually active in the past 4 weeks [No, yes] and ever experienced a terminated pregnancy [No, yes].

### Statistical analysis

Data analysis was performed using STATA version 14. Data was set to account for the complex nature of survey design through application of weights, primary sampling unit (cluster) and strata. To evaluate the trends in modern contraceptive use, descriptive analysis was done separately, stratified across the three surveys phases; 2004–2010 (phase 1), 2010–2016 (phase 2) and 2004–2016 (phase 3).

Multivariable Poisson-based decomposition models were used to determine the changes in modern contraceptive use and associated factors across survey years. The change in modern contraceptive use was explained by changes in the population structure (endowments) as well as changes in the effects of the characteristics (coefficients). The Blinder-Oaxaca decomposition [17] was chosen as it allows to analyse the outcome of two different groups. This method allows to display the contribution of each variable to the overall difference in characteristics or the effects of characteristics. Therefore, at any instance of time, this method allows to compare modern contraceptive use between two time periods (2004/2005 and 2015/2016). Furthermore, it partitions the difference in modern contraceptive use between the time periods into components that are attributable to changes in composition, changes in effects and the interaction between them. All associations were considered statistically significant when the p-value was less than 0.05.

### Ethical considerations

Ethical approval was obtained from Kilimanjaro Christian Medical University Research and Ethics Review Committee (CREC no. 2395). Permission was granted to download and use the data from DHS Program/ICF International. Data was used solely for the purpose of the current study. Detailed information about the methodology and ethical considerations for the DHS program are published in the Tanzania Demographic and Health survey reports [9,18,19].

## Results

### Background characteristics of study participants

Background characteristics of the study participants are shown in Table 1. The proportion of women aged 25–34 years decreased across the survey years from 37.5% in 2004/2005, to 34.3% in 2010 and 32.4% in 2015/2016. The proportion of women who resided in rural areas remained relatively the same at 71.2% in 2004/2005 and 71.4% in 2010 and then decreased to 64.1% in 2015/2016. Partner education levels increased across the surveys where those having secondary education and above increased from 10.3% in 2004/2005, to 12.2% in 2010 and 19.0% in 2015/2016. The proportion of women who reported their last pregnancy as mistimed increased across the three surveys starting from 19.1% in 2004/2005 to 24.6% in 2010 and further increased to 29.6% in 2015/2016.

**Table 1:** Background characteristics (weighted) of study participants in the TDHS 2004/2005, 2010 and 2015/2016 surveys (N = 26,141).

Variable	Survey year		
	2004/2005 (n = 7,891)	2010 (n = 7,767)	2015/2016 (n = 10,483)
**Age (5 years)**			
15–24	31.1	29.3	31.2
25–34	37.5	34.3	32.4
35–49	31.4	36.4	36.4
**Male–female age difference**			
Woman older	3.4	3.9	4.8
Same age	2.5	3.1	3.6
Man older ≤9 years	64.4	65.6	66.2
Man older >9 years	29.7	27.4	25.4
**Zone**			
Western	9.1	8.6	9.3
Northern	11.4	12.6	11.6
Central	10.4	10.2	10.1
Southern highlands	7.3	8.6	6.4
Southern zone	6.1	6.6	5.8
South West Highlands	9.8	8.6	9.5
Lake zone	26.3	25.3	25.8
Eastern	17.1	17.1	19.2
Zanzibar	2.4	2.5	2.3
**Residence**			
Urban	28.8	28.6	35.9
Rural	71.2	71.4	64.1
**Woman’s education level**			
No education	26.0	21.0	16.2
Primary education	66.7	66.7	64.1
Secondary and above	7.3	12.2	19.7
**Partner’s education level**			
No education	18.9	15.8	12.0
Primary education	70.9	72.0	69.0
Secondary and above	10.3	12.2	19.0
**Number of living children**			
None	14.0	13.3	14.8
1–2	36.8	35.0	36.4
3–4	25.8	27.0	25.5
5 or more	23.4	24.6	23.2
**Wealth Categories**			
Poor	37.1	36.6	34.7
Middle	18.8	19.4	17.9
Rich	44.1	44.0	47.3
**Heard FP in media during past months**			
No	40.6	45.7	31.2
Yes	59.4	54.3	68.8
**Last Pregnancy**			
Wanted	74.4	70.3	65.0
Mistimed	19.1	24.6	29.6
Unwanted	6.5	5.1	5.4
**Family size concordance**			
Both want same	43.3	41.4	40.3
Husband wants more	29.7	26.2	26.0
Husband wants fewer	4.7	5.4	6.3
Don’t know	22.3	27.0	27.4
**Visited by FP worker in household**			
No	96.7	95.4	96.1
Yes	3.3	4.6	3.9
**Sexually active in past 4 weeks**			
No	35.5	34.7	36.5
Yes	64.5	65.3	63.5
**Ever terminated pregnancy**			
No	80.4	80.3	81.7
Yes	19.6	19.7	18.3

### Trends in modern contraceptive use

**Fig 2:**
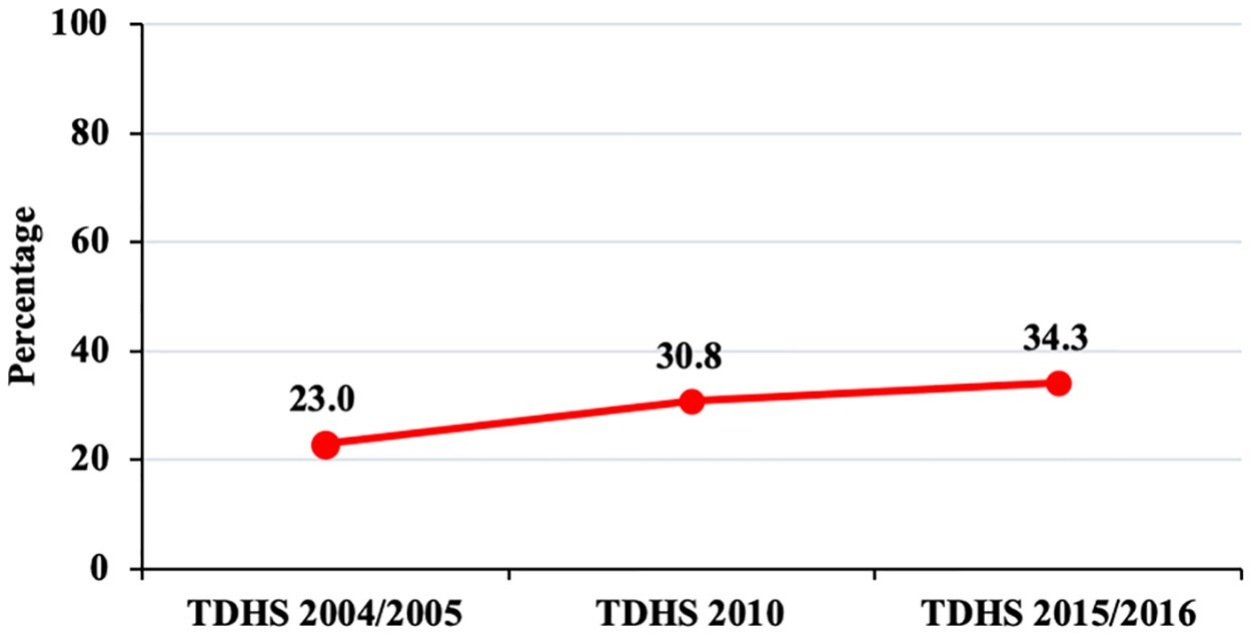
Trends in modern contraceptive use among women aged 15–49 years in the past 10 years, Tanzania Demographic and Health Surveys, 2004/2005–2015/2016 (N = 26,141).

Modern contraceptive use has increased steadily from 23.0% in 2004/2005, 30.8% in 2010 and 34.3% in 2015/2016 (Fig 2). The trend period of modern contraceptive use was categorized into 3 phases; 2004–2010 (Phase 1), 2010–2016 (Phase 2) and 2005–2016 (Phase 3/overall phase) in order to detect the differences in modern contraceptive use over time. Overall, use of modern contraceptives increased by 11.3 percentage points. The largest increase in modern contraceptive use was observed in phase 1, at 7.8 percentage point increase compared with a 3.5 percentage point increase during phase 2.

**Fig 3:**
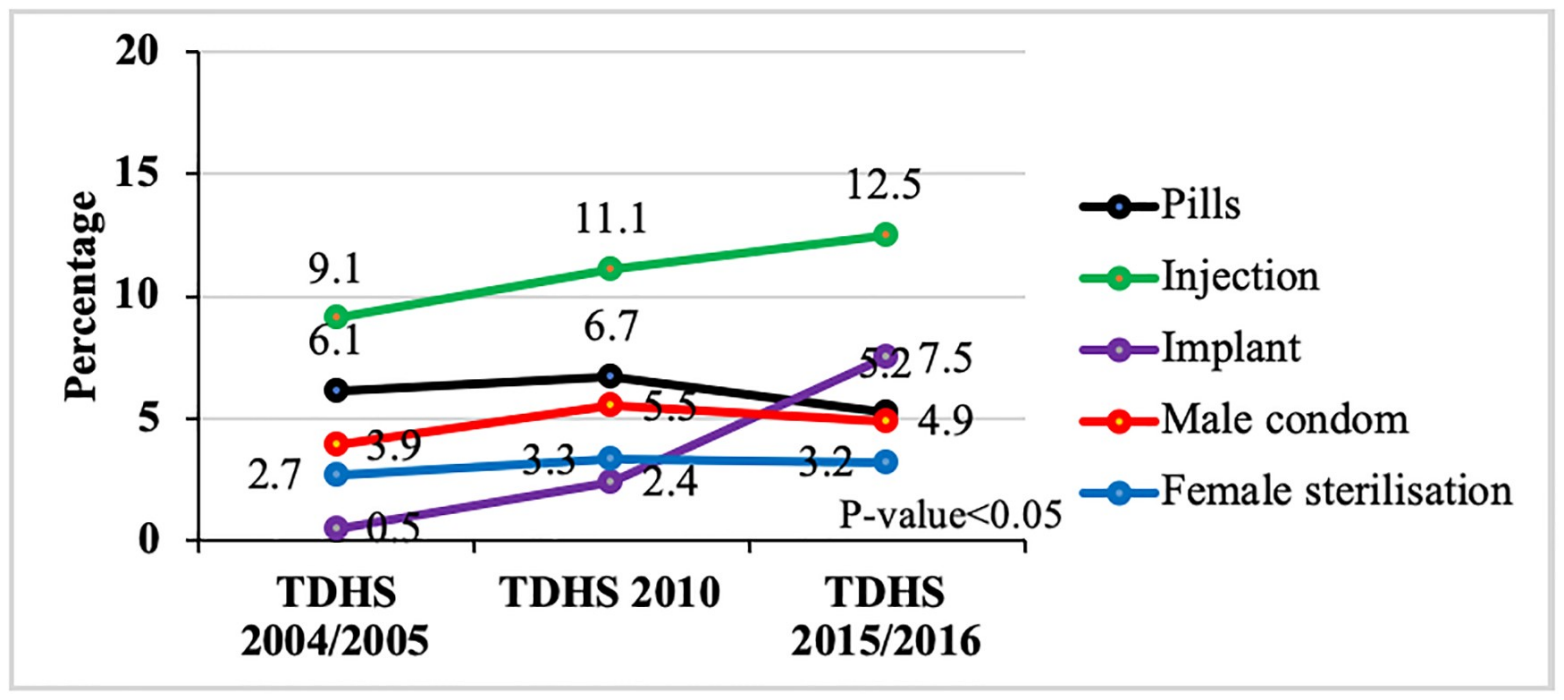
Trends in modern contraceptive use among women aged 15–49 years by method types in the past 10 years, Tanzania Demographic and Health Surveys, 2004/2005–2015/2016.

There was also a variation in modern contraceptive use according to method types (Fig 3). The proportion of women who used contraceptive pills increased from 6.1% in 2004/2005 to 6.7% in 2010 and then decreased to 5.2% in 2015/2016. The proportion of women who used implants increased from 0.5% in 2004/2005 to 2.4% in 2010 and further to 7.5% in 2015/2016 surveys respectively. The number of women who used injections also increased across the 3 surveys with an overall increase of 3.4 percentage points.

Trends in modern contraceptive use varied according to women’s characteristics across the surveys. There was an increase in modern contraceptive use across age groups. The greatest increase occurred among women aged 25–34 years at an overall 13.0 percentage point increase from 27.6% in 2004/2005 to 40.6% in 2015/2016. Among these women, the largest increase was observed during phase 1 at 7.3 percentage point increase compared with a 5.7 percentage increase in phase 2. Among couples of the same age, the largest increase was observed during phase 2 at 11.0 percentage point increase compared with a 6.5 percentage point increase during phase 1 (Table 2). The largest increase in modern contraceptive use among women living in rural areas occurred during phase 1 at 11.2 percentage point increase compared with a 4.0 percentage point increase during phase 2.

**Table 2:** Trends in modern contraceptive use among women aged 15–49 years by selected characteristics, in the TDHS 2004/2005, 2010 and 2015/2016 surveys (N = 26,141).

Characteristic	TDHS 2005/2006^Ω^	TDHS 2010^Ψ^	TDHS 2015/2016^ω^	Percentage point difference in modern contraceptive use		
				Phase I ^a^	Phase II ^b^	Phase III ^c^
**Age (5 years)**						
15–24	20.5	29.1	29.5	8.6	0.4	9.0
25–34	27.6	34.9	40.6	7.3	5.7	13.0
35–49	20.1	28.2	32.8	8.1	4.6	12.7
**Male–female age difference**						
Woman older	16.1	22.8	32.1	6.7	9.3	16.0
Same age	24.7	31.2	42.2	6.5	11.0	17.5
Man older ≤ 9 years	25.1	33.7	37.9	8.6	4.2	12.8
Man older > 9 years	19.9	27.1	31.8	7.2	4.7	11.9
**Residence**						
Urban	36.3	35.6	36.9	-0.7	1.3	0.6
Rural	17.7	28.9	32.9	11.2	4.0	15.2
**Woman’s education level**						
No education	9.7	19.9	26.6	10.2	6.7	16.9
Primary education	26.7	33.3	36.4	6.6	3.1	9.7
Secondary and above	36.4	35.9	34.1	-0.5	-1.8	-2.3
**Partner’s education level**						
No education	10.5	17.4	22.2	6.9	4.8	11.7
Primary education	23.9	32.2	38.4	8.3	6.2	14.5
Secondary and above	42.5	39.8	37.2	-2.7	-2.6	-5.3
**Heard FP through tv, radio or newspaper in last months**						
No	14.4	24.7	29.7	10.3	5.0	15.3
Yes	28.9	35.9	36.4	7.0	0.5	7.5
**Number of living children**						
None	13.0	21.6	17.0	8.6	-4.6	4.0
1–2	25.6	31.6	36.3	6.0	4.7	10.7
3–4	27.2	35.4	41.2	8.2	5.8	14.0
5 or more	20.4	29.6	34.7	9.2	5.1	14.3
**Wealth Categories**						
Poor	13.8	23.7	27.3	9.9	3.6	13.5
Middle	18.4	26.8	37.9	8.4	11.1	19.5
Rich	32.8	38.5	38.2	5.7	-0.3	5.4
**Last Pregnancy**						
Wanted	24.2	33.4^Φ^	38.5^Φ^	9.2	5.1	14.3
Mistimed	27.8	33.4	39.4	5.6	6.0	11.6
Unwanted	30.6	42.7	39.8	12.1	-2.9	9.2
**Family size concordance**						
Both want same	27.8	35.5	39.5	7.7	4.0	11.7
Husband wants more	14.9	23.1	28.9	8.2	5.8	14.0
Husband wants fewer	23.6	31.7	39.1	8.1	7.4	15.5
Don’t know	15.0	22.4	28.2	7.4	5.8	13.2
**Visited by FP worker in household**						
No	22.7	30.5^Φ^	33.3	7.8	2.8	10.6
Yes	31.4	36.3	60.5	4.9	24.2	29.1
**Sexually active in past 4 weeks**						
No	13.0	18.5	21.5	5.5	3.0	8.5
Yes	28.5	37.4	41.7	8.9	4.3	13.2
**Ever had a terminated pregnancy**						
No	24.1	31.9	34.5^Φ^	7.8	2.6	10.4
Yes	18.6	25.9	33.7	7.3	7.8	15.1
**Total**	**23.0**	**30.8**	**34.3**	**7.8**	**3.5**	**11.3**

^Ω^ n = 7,891;

^Ψ^ n = 7,767;

^ω^ n = 10,483;

^a^ 2005–2010;

^b^ 2010–2015;

^c^ 2005 and 2015;

^Φ^ p-value > 0.05

### Factors associated with changes in modern contraceptive use

The results of the Poisson decomposition regression analysis for factors associated with changes in modern contraceptive use between 2004/2005 and 2015/2016 are presented in Table 3. We found that 12.5% of the overall percentage change in modern contraceptive use was attributable to the difference in characteristics (*compositional changes*). These included partner’s education levels, wealth categories, exposure to family planning messages in the media as well as recent sexual activity. An increase in the proportion of women whose partners attained secondary level of education and above (Table 1) resulted in a significant positive contribution on modern contraceptive use between 2004/2005 and 2015/2016 (4.17%, p-value: 0.001).

**Table 3:** Poisson decomposition of changes in modern contraceptive use among women aged 15–49 years in Phase 3 (N = 26,141).

Modern contraceptive use	Due to differences in characteristics (E)		Due to differences in coefficients (C)	
	Coeff.	%	Coeff.	%
**Age (5 years)**				
15–24	1.0		1.0	
25–34	-0.0007	-0.5085	0.0046	3.5751
35–49	-0.0013 *	-1.0216	0.0007	0.5459
**Male–female age difference**				
Woman older	1.0		1.0	
Same age	0.0002	0.1887	0.0006	0.4863
Man older ≤ 9 years	-0.00004	-0.0316	-0.0283	-21.967
Man older > 9 years	0.0010	0.7567	-0.0162	-12.530
**Zone**				
Western	1.0		1.0	
Northern	0.0001 ***	0.0394	-0.0111 **	-8.5925
Central	0.00003 ***	0.0269	-0.0065	-5.0765
Southern highlands	-0.0008 ***	-0.6558	-0.0049 *	-3.8294
Southern zone	-0.0007 ***	-0.5305	-0.0023	-1.7935
South West Highlands	-0.0024 ***	-1.8956	-0.0053	-4.0831
Lake zone	-0.00002	-0.0190	0.0081	6.3148
Eastern	0.0023 ***	1.7765	-0.0075	-5.8302
Zanzibar	-1.44*10^−06^ ***	-0.0011	-0.0014	-1.0881
**Residence**				
Urban	1.0		1.0	
Rural	-0.0020 *	-1.5477	0.0568 ***	44.071
**Woman’s education level**				
No education	1.0		1.0	
Primary education	-0.0005	-0.4082	-0.0535 ***	-41.517
Secondary and above	0.0023	1.8058	-0.0039 *	-3.0405
**Partner’s education level**				
No education	1.0		1.0	
Primary education	-0.0031 ***	-2.3787	0.0041	3.1479
Secondary and above	0.0054 **	4.1710	-0.0016	-1.2368
**Wealth Categories**				
Poor	1.0		1.0	
Middle	-0.0008 ***	-0.6227	0.0066	5.1288
Rich	0.0002 ***	0.1268	0.0076	5.8683
**Heard of FP in media during past months**				
No	1.0		1.0	
Yes	0.0016 *	1.2299	-0.0204 *	-15.798
**Last Pregnancy**				
Wanted	1.0		1.0	
Mistimed	0.0008	0.6050	0.0021	1.6242
Unwanted	-0.0003 *	-0.2646	-0.0006	-0.4316
**Family size concordance**				
Both want same	1.0		1.0	
Husband wants more	0.0008 *	0.5960	0.0081	6.2923
Husband wants fewer	0.0002	0.1323	0.0025	1.9697
Don’t know	-0.0016 **	-1.2267	0.0064	4.9382
**Visited by FP worker in household**				
No	1.0		1.0	
Yes	0.0005 ***	0.3585	0.0016	1.2432
**Sexually active in past 4 weeks**				
No	1.0		1.0	
Yes	0.0087 ***	6.7753	-0.0230	-17.861
**Ever terminated pregnancy**				
No	1.0		1.0	
Yes	-8.70*10^−07^	-0.0007	0.0092 **	7.1271
Constant			0.1802 **	139.80
**Total**	0.0162 ***	12.543	0.1127***	87.457

* Significant at p<0.05;

** Significant at p<0.01;

*** Significant at p<0.001

Similarly, an increase in the proportion of women who were exposed to family planning messages in mass media such at TV, radio and newspapers between 2004/2005 and 2015/206 surveys (Table 1) led to significant positive contribution in the changes of modern contraceptive use (1.22%, p-value: 0.028) (Table 3). Recent sexually activity also accounted for positive changes in modern contraceptive use. We noted a slight decrease in the proportion of women who reported being sexually active in the past 4 weeks during the 2015/2016 survey (Table 1) leading to a significant positive contribution (6.77%, p-value: <0.001) on modern contraceptive use (Table 3). A decrease in the proportion of women whose husbands wanted more children (Table 1), resulted in a significant positive contribution (0.6%, p-value: 0.042) on modern contraceptive use (Table 3).

After controlling for compositional factors above, most (87.45%) of the increase in modern contraceptive use was contributed by the changes in the effects of characteristics (*changes in coefficients*). These include area of residence and experience of a terminated pregnancy (Table 3). These factors showed a significant effect on the observed positive change in modern contraceptive use between 2004/2005 and 2015/2016 surveys. We also found that, nearly half (44.07%) of the increase in modern contraceptive use during the study period was contributed by changes in modern contraceptive use among women residing the rural areas (p-value: <0.001). Furthermore, women who experienced a terminated pregnancy had a significant positive contribution to the observed increase in modern contraceptive use compared to their counterparts during the study period (7.12%, p-value: 0.009).

## Discussion

The present study demonstrated that modern contraceptive use in Tanzania has increased yearly for the past 10 years. This finding is consistent with the United Nations report and the DHS studies from Ghana and Zambia which reported an increase of modern contraceptive use with time [20–22]. The increase of mCPR in Tanzania may be explained by the government efforts/interventions to reposition family planning as a national priority in recognition of its importance in accelerating progress towards attainment of Sustainable Development Goals (SDGs). These include the re-launching of the Green Star media campaign in 2013, updating the NFPRA, formulation of the One Plan II as well as FP2020 commitments [14–16,23]. These efforts collectively may have contributed to the observed increase in mCPR during the last decade in Tanzania.

In the present study, about 13% of the change in modern contraceptive use among women of reproductive age was due to differences in characteristics. This is lower compared to findings from Cameroon (69%), Rwanda (17%) and Ethiopia (34%) [10,11,24]. The difference in the observed changes in mCPR could be explained by the differences in population structure and characteristics between countries. This implied that, a significant change in mCPR arises when the structure of the population changes according to important variables.

We found that, an increase in proportion of women whose partners attained secondary and above education level had a significant positive contribution on modern contraceptive use. This result is contrary to a study in Ethiopia which found no significant contribution of partner’s education level on modern contraceptive use [10]. Our finding suggests that partners play a significant role in decisions regarding use of modern contraceptives. Therefore, education of men could be an important factor to focus on in order to further increase mCPR in Tanzania.

The decline in the proportion of husbands who wanted more children than their wives contributed significantly to the increase in modern contraceptive use in Tanzania during the past 10 years. This finding may reflect the open discussions about family size between couples as well as the possible role of women empowerment [6]. Empowered women are more likely to use modern contraceptives. This highlights the importance of couple’s joint decisions pertaining to sexual reproductive health matters especially modern contraceptive use.

In this study, an increase in the proportion of women who heard of family planning in the media had a significant positive contribution to the increase of modern contraceptive use during the last decade. A similar finding was reported in Rwanda [11]. Media such as TV, radio and newspapers remain a powerful tool to reach a large number of women and provide information regarding modern contraceptives. There is a need for intensified efforts on awareness creation on modern contraceptives through media. Our finding may also reflect the effects of the Green Star media campaign in 2013 which further helped to get information regarding modern contraceptives to both women and men of reproductive age in Tanzania [23].

Majority of the increase in modern contraceptive use was contributed by changes in effects of characteristics. This was consistent with previous reports from Rwanda and Ethiopia [10,11]. This suggests that after controlling for the differences in characteristics, the main changes in modern contraceptive use were attributed to changes in effects of certain variables that had significant influence on modern contraceptive use in Tanzania.

This study found that woman’s education level had a negative contribution on the changes in modern contraceptive use. Women with any form of education had a lower change in modern contraceptive use similar to findings in Rwanda [11]. This implies that even though education is known to have a positive association with modern contraceptive use, family planning initiatives may have reached even women with no education.

Similar to a study in Ethiopia [10], 44% of the increase in modern contraceptive use in this study was due to changes of contraceptive use among women residing in rural areas. Although the difference in mCPR between rural and urban areas was not large (36.9% vs 32.9%), the largest increase in mCPR was observed among women residing in rural areas. This could be explained by the national efforts invested to increase modern contraceptive use in rural areas through various commitments such as publicizing the relaunch of the Green Star campaign (as earlier indicated), training of community health workers in order to increase FP service provision at the community level as well as engagement of religious leaders to promote family planning [15].

### Strengths and weakness

This study had a number of strengths. The utilization of data from the Tanzania Demographic and Health survey provided an opportunity for generalization of our findings due to a large sample size and statistical power to make conclusions on modern contraceptive use. The application of decomposition analysis also allowed to account for changes in the population structure and dynamics over time.

Although this study highlighted important findings to support family planning programs in Tanzania, we could not examine the effect of some important variables such as use of health insurance, partner awareness of contraceptive use, number of sexual partners, religion and family planning services availability as well quality. These variables have been reported to influence modern contraceptive use. These variables were not collected in all the three surveys hence could not be used in the analysis. Therefore, the effect of these variables on our results were not quantified.

## Conclusion

Although modern contraceptive use has steadily increased in Tanzania, we are far from reaching the national targets. Changes in modern contraceptive use during the last decade in Tanzania were attributed to increasing partner education, rich wealth category, FP media exposure, residence, family size concordance and experience of a terminated pregnancy. Designing of interventions with emphasis on the role of media campaigns will be a gateway to increase mCPR in Tanzania. Campaigns will keep the community informed and updated on the potential benefits of modern contraceptives. Furthermore, emphasis should also be given to women who experienced termination of pregnancy as an entry point to encourage modern contraceptive use.

## Supporting information

S1 Data (ZIP)

## Abbreviations

HIV: Human Immunodeficiency virus
mCPR: Modern contraceptive prevalence rate
TDHS: Tanzania Demographic and Health Surveys
